# A treatment option for rectal gastrointestinal stromal tumor: endoscopic submucosal dissection combined with endoscopic hand-suturing

**DOI:** 10.1055/a-2523-2684

**Published:** 2025-02-18

**Authors:** Lizhou Dou, Angshu Cai, Shibo Song, Guiqi Wang, Shun He

**Affiliations:** 1 Endoscopy, National Cancer Center/National Clinical Research Center for Cancer/Cancer Hospital, Chinese Academy of Medical Sciences and Peking Union Medical College, Beijing, China; 2 26447 Endoscopy Center, Peking University First Hospital, Beijing, China

Rectal gastrointestinal stromal tumors (GISTs) are very rare, accounting for about 5% of all GISTs 1 . Treatment for a rectal GIST should be carefully selected to avoid postoperative complications and impairment of anal sphincter function 2 3 . Recently, several cases of endoscopic resection for rectal GISTs have been reported, but there is a lack of reports on strategies for defect closure 4 5 . We report a novel method for treating a rectal GIST via a combination of endoscopic hand-suturing (EHS) with endoscopic submucosal dissection (ESD).

A 64-year-old man presented to our center with a rectal mass located 3 cm from the anal verge. Magnetic resonance imaging (MRI) and endoscopic ultrasound (EUS) identified a submucosal tumor measuring 1.2 × 1.0 cm in the lower rectum ( Fig. 1 ). After a multidisciplinary discussion, the tumor was considered to be a rectal GIST. Subsequently, having been fully informed of the condition and its associated risks, the patient chose to undergo ESD to treat the lesion and clarify the diagnosis.

**Fig. 1:**
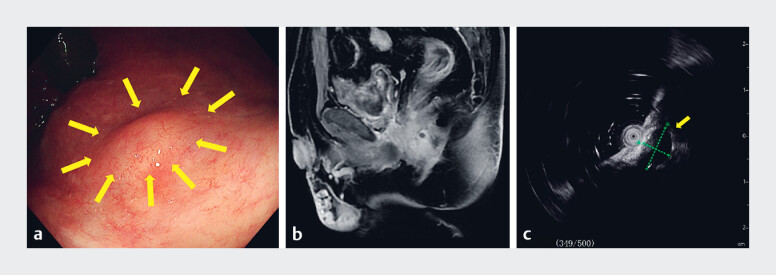
The preoperative examination findings were: **a** on colonoscopy, a submucosal tumor (arrow) located 3 cm from the anal verge; **b** on magnetic resonance imaging, the tumor was found to be situated in the lower rectum without invasion of adjacent organs; **c** on endoscopic ultrasound, the tumor (arrow) was hypoechoic and measured 1.2 × 1.0 cm.

The steps of ESD were performed strictly according to protocols and the tumor was removed en bloc ( Fig. 2 **a, b** ). After resection, a significant depression was observed in the artificial ulcer, accompanied by damage to the muscularis propria layer ( Fig. 2 **c** ). To prevent postoperative perforation, we performed endoscopic hand-suturing to close the defect and secured the suture tail with a clip after removing the needle ( Fig. 3 ; Video 1 ). The ESD and endoscopic hand-suturing times were 51 and 53 minutes, respectively. The patient was discharged on postoperative day 4, without any adverse events having occurred. Histology confirmed that the tumor was a very low risk GIST and had been completely resected. Colonoscopy during the third postoperative month showed a healed scar without any signs of recurrence ( Fig. 4 ).

**Fig. 2:**
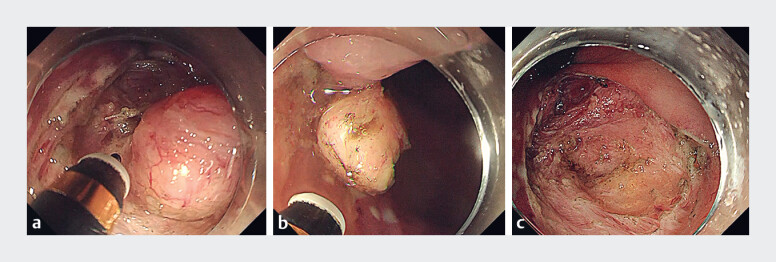
Endoscopic images during resection of a rectal gastrointestinal stromal tumor (GIST) by endoscopic submucosal dissection (ESD) showing: **a** dissection of the submucosa; **b** the tumor completely removed with the capsule remaining intact; **c** the defect after ESD, with accompanying damage to the muscularis propria layer.

**Fig. 3:**
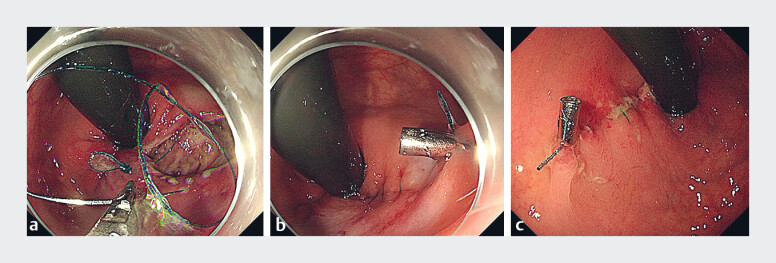
Endoscopic images after the endoscopic submucosal dissection of the rectal lesion showing: **a** closure of the defect using an absorbable barbed suture with needle (VLOCL0803; Covidien, Mansfield, Massachusetts, USA) and a needle holder originally designed by our team (entrusted manufacturer: Vedkang, Jiangsu, China); **b** the suture tail secured with a clip after closure of the defect; **c** the appearance 3 days after closure of the defect by endoscopic hand-suturing.

**Fig. 4:**
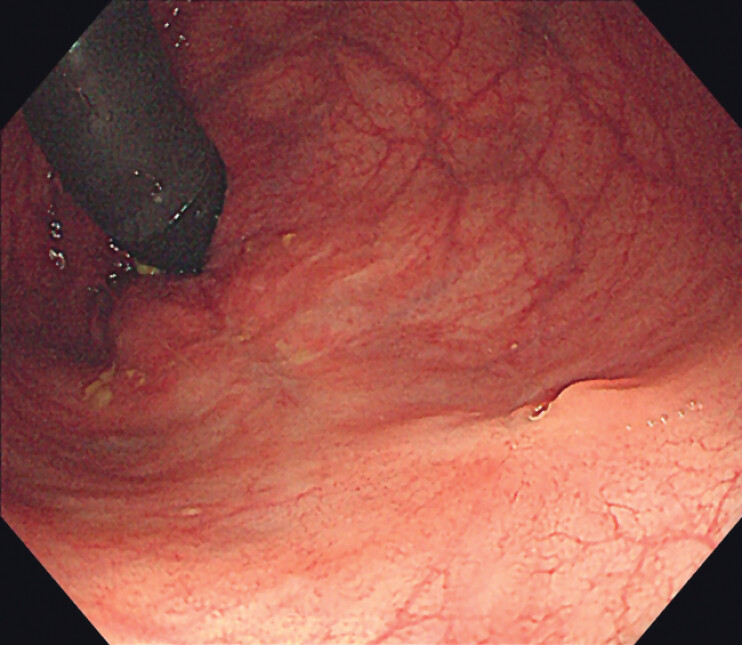
Follow-up colonoscopic image 3 months after the procedure showing a well-healed defect with fibrotic scar formation.

Closure of the defect, which showed muscularis propria damage, after complete removal of a rectal gastrointestinal stromal tumor by endoscopic submucosal dissection using endoscopic hand-suturing.Video 1

The use of ESD combined with endoscopic hand-suturing for treatment of a rectal GIST has not been previously reported. The application of endoscopic hand-suturing in this case successfully avoided postoperative adverse events, demonstrating the safety and feasibility of this novel method for treating rectal GISTs.

Endoscopy_UCTN_Code_TTT_1AQ_2AK

## Notes

Endoscopy E-Videos https://eref.thieme.de/e-videos
*E-Videos* is an open access online section of the journal *Endoscopy* , reporting on interesting cases and new techniques in gastroenterological endoscopy. All papers include a high-quality video and are published with a Creative Commons CC-BY license. Endoscopy E-Videos qualify for HINARI discounts and waivers and eligibility is automatically checked during the submission process. We grant 100% waivers to articles whose corresponding authors are based in Group A countries and 50% waivers to those who are based in Group B countries as classified by Research4Life (see: https://www.research4life.org/access/eligibility/ ). This section has its own submission website at https://mc.manuscriptcentral.com/e-videos .

## References

[1] Tran T Davila JA El-Serag HB The epidemiology of malignant gastrointestinal stromal tumors: an analysis of 1,458 cases from 1992 to 2000 Am J Gastroenterol 2005 100 162 168 10.1111/j.1572-0241.2005.40709.x 15654796

[2] Kim SJ Jung Y Hong R Successful endoscopic resection of a rectal gastrointestinal stromal tumor larger than 5 cm Korean J Gastroenterol 2021 78 235 239 34697278 10.4166/kjg.2021.097 PMC12286440

[3] Kameyama H Kanda T Tajima Y Management of rectal gastrointestinal stromal tumor Transl Gastroenterol Hepatol 2018 3 8 10.21037/tgh.2018.01.08 29552659 PMC5847932

[4] Ishida T Furumatsu K Kado T Endoscopic submucosal dissection of a rectal gastrointestinal stromal tumor close to the dentate line Dig Endosc 2020 32 e49 e51 10.1111/den.13605 31925831

[5] Mavrogenis G Maurommatis E Koumentakis C Submucosal tunneling endoscopic resection for rectal gastrointestinal stromal tumor Endoscopy 2023 55 E619 E620 10.1055/a-2055-1102 37040883 PMC10089791

